# Influence of the Inclination Angle of 3D-Printed Inconel Alloy 718 on Its Corrosion Resistance

**DOI:** 10.3390/ma19061126

**Published:** 2026-03-13

**Authors:** Aleksandra Iwańczak, Katarzyna Skibińska, Krzysztof Żaba, Maciej Balcerzak, Konrad Wojtaszek, Sławomir Kąc, Piotr Żabiński

**Affiliations:** 1 Department of Metal Working and Physical Metallurgy of Non-Ferrous Metals, Faculty of Non-Ferrous Metals, AGH University of Krakow, al. Adama Mickiewicza 30, 30-059 Krakow, Poland; aiwanczak@agh.edu.pl (A.I.); krzyzaba@agh.edu.pl (K.Ż.); balcerzak@agh.edu.pl (M.B.); 2 Department of Physical Chemistry and Metallurgy of Non-Ferrous Metals, Faculty of Non-Ferrous Metals, AGH University of Krakow, al. Adama Mickiewicza 30, 30-059 Krakow, Poland; kskib@agh.edu.pl (K.S.); kwojtasz@agh.edu.pl (K.W.); 3 Faculty of Metals Engineering and Industrial Computer Science, AGH University of Krakow, al. Adama Mickiewicza 30, 30-059 Krakow, Poland; slawomir.kac@agh.edu.pl

**Keywords:** additive manufacturing, PBF-LB/M, Inconel 718, inclination angle, corrosion, potentiodynamic polarization

## Abstract

This study aimed to investigate the influence of the synthesis parameters on the corrosion resistance of 3D-printed Inconel 718 components. Samples were fabricated using laser powder bed fusion (PBF-LB/M) with different angles of inclination. Corrosion tests were conducted by immersion for 1000 h in a 3.5% aqueous NaCl solution at 20 °C and 45 °C, and by the potentiodynamic polarization measurements. Detailed analysis of changes in morphology, chemical composition, and roughness of 3D prints was performed using scanning electron microscopy, combined with energy-dispersive X-ray spectroscopy, and optical profilometry. To quantify the dissolution of alloy components during the long-term measurements, the post-corrosion solutions were analyzed using microwave plasma–atomic emission spectroscopy. The obtained results demonstrate that inclination angle significantly affects corrosion rate and electrochemical kinetics, with measurable differences in mass loss, Icorr values, and surface degradation morphology observed between orientations. The findings indicate that build orientation governs microstructural anisotropy and surface characteristics, which in turn influence corrosion susceptibility. The novelty of this work lies in the systematic and multi-method evaluation of inclination angle as an independent structural parameter controlling corrosion kinetics in PBF-LB/M-fabricated Inconel 718, providing new insight into structure–corrosion relationships in additively manufactured nickel-based superalloys.

## 1. Introduction

Additive manufacturing (AM) technologies have significantly transformed the production of advanced metallic components, enabling the fabrication of complex geometries with tailored microstructures and enhanced functional properties. Among various AM techniques, laser powder bed fusion (LPBF) has emerged as one of the most promising methods for manufacturing high-performance nickel-based superalloys, particularly Inconel 718 (IN718), which is widely applied in aerospace, energy, and high-temperature engineering due to its excellent mechanical strength, corrosion resistance, and thermal stability [1,2,3,4].

The LPBF process is characterized by rapid melting and solidification, resulting in extremely high cooling rates and steep thermal gradients. These conditions lead to the formation of non-equilibrium microstructures, including columnar grains, cellular dendritic structures, and pronounced microsegregation of alloying elements [5,6,7,8]. Consequently, the microstructure of LPBF-fabricated IN718 differs significantly from that of conventionally processed materials, which strongly affects mechanical properties, anisotropy, and fracture behavior [9,10,11,12].

The mechanical performance of additively manufactured IN718 is closely related to its microstructural features and phase composition. The strengthening mechanisms in this alloy are primarily associated with γ′ and γ″ precipitates, while the presence of δ and Laves phases may deteriorate mechanical properties [13,14,15]. Numerous studies have demonstrated that heat treatment strategies, including solution treatment and aging, can effectively modify the microstructure, enhance precipitation hardening, and improve tensile strength and ductility of LPBF-fabricated IN718 [16,17,18]. Moreover, post-processing methods such as hot isostatic pressing (HIP) have been shown to reduce porosity and improve fatigue performance [19,20,21].

In addition to additive manufacturing, nickel-based superalloys are widely investigated in the context of forming and deformation processes. Recent studies on sheet forming of Inconel alloys using flexible tools and numerical modeling have provided valuable insights into deformation mechanisms and material behavior under complex loading conditions [22,23]. These investigations contribute to a broader understanding of the mechanical response of nickel-based superalloys and complement research on additively manufactured materials.

The influence of process parameters and build orientation on the mechanical behavior and fracture mechanisms of LPBF-fabricated IN718 has been extensively studied. Experimental investigations have revealed that build orientation significantly affects tensile properties, anisotropy, and fracture characteristics of additively manufactured components [24,25,26]. Detailed fracture analyses have further demonstrated the relationship between microstructural features, defect distribution, and failure mechanisms in LPBF-produced IN718 [27,28,29].

Beyond mechanical and microstructural aspects, technological and sustainability-related issues have gained increasing attention in additive manufacturing research. Powder reuse, energy consumption, and surface finishing processes significantly influence the economic and environmental viability of LPBF technology [30,31,32]. Integrated approaches combining experimental investigations and numerical modeling have been proposed to optimize manufacturing efficiency and improve the overall performance of additively manufactured components [33].

Despite substantial progress in understanding the LPBF processing of IN718, several research gaps remain. The complex interplay between process parameters, thermal history, microstructural evolution, mechanical performance, and corrosion resistance has not yet been fully elucidated. Furthermore, the combined effects of additive manufacturing and subsequent forming or deformation processes on the mechanical behavior of nickel-based superalloys require further systematic investigation. Therefore, comprehensive studies integrating experimental and numerical approaches are essential for establishing robust process–structure–property relationships and enabling reliable industrial applications of LPBF-fabricated Inconel 718.

IN 718 is applied in the aerospace and petrochemical industries due to its mechanical properties and oxidation resistance at elevated temperatures [34]. However, when applied in a gas turbine, it suffers from the hot corrosion triggered by salts like NaCl, Na_2_SO_4_, and V_2_O_5_ in the combustion chamber [35]. The literature review shows that NaCl coating causes most damage due to the small size of Cl^−^ ions and their great penetrating power [36]. Cr- and Mo-enriched composite oxide layers are crucial to ensure the integrity of the passive film protecting against corrosion [37]. Additionally, with smaller surface roughness, the corrosion surface area is reduced [38,39]. L.C.M. Valle and others [40] proposed aging of alloy 718 at 800 °C for 24 h to improve its corrosion resistance due to the presence of a created passive Al-rich oxide layer. An aluminide coating can also be deposited on the IN718 to protect the alloy against an environment composed of air, NaCl, and water vapor at 750 °C [41]. Due to the specific microstructure, which is fine and heterogeneous with partially melted particles, and high porosity, 3D-printed metals and alloys can show worse corrosion resistance than their cast counterparts. Inconel 718 alloy produced using the selective laser melting (SLM) method shows worse corrosion resistance in 3.5 wt. % NaCl at room temperature than the commercially rolled alloy [42]. It is connected to the composition of a porous passive film formed on SLM alloy, which contains more NiO and less Cr_2_O_3_ than the film formed on the rolled one. The presence of Ni oxides is expected, as it appeared on the surface of Ni structures after the room temperature corrosion tests in NaOH [43]. Pitting corrosion on 316L stainless steel produced by laser powder bed fusion (LPBF) was observed after 35 days of immersion in 3.5 wt. % NaCl [44]. It was found that it was caused by Mn-rich silicate slags that introduced cracks or surface oxide heterogeneities. In the case of spark plasma sintered stainless steel 316L matrix composites with zirconium diboride, the presence of the ceramic ZrB_2_ phase changes the corrosion resistance of the materials due to porosity, which affects the corrosion mechanism [45]. Composites cannot achieve complete passivation due to the penetration of sulfuric acid into the pores. The influence of the synthesis parameters on the corrosion rate of steel M300 fabricated by direct metal laser sintering (DMLS) in 3.5 wt. % NaCl was analyzed [46]. With the increase in laser power, the corrosion resistance of the 3D-printed components generally decreased. Mechanical polishing did not improve the corrosion resistance of the alloy. Irrelevant of surface treatment, the corrosion rate increased significantly at higher temperatures. Similar research was performed for the maraging steel M350, produced by the LPBF method [47]. In this case, higher laser power (≥120 W) reduced corrosion rates at room temperature, while lower power (80–100 W) performed better at elevated temperatures (45 °C). Additionally, samples were mechanically polished. Also in this case, the surface treatment did not improve corrosion resistance. But, in some cases, it increased corrosion rates, likely due to stress redistribution and exposure of subsurface defects. For Inconel alloy 718 synthesized using the SLM technique, corrosion resistance can be improved by application of a scanning space of near or equal to the laser diameter, which helps to avoid unmelted powder or spheroidization defects [48]. J. Zhang and others noticed that the products of hot corrosion of IN718 produced by the laser metal deposition (LMD) technique consist of two layers, with Ni_3_S_2_ in the inner layer and mainly Ni_2_CrO_4_, Cr_2_O_3_, and Fe_3_O_4_ in the outer layer [49]. The application of the heat treatment can improve the resistance to corrosion of the alloy produced by additive manufacturing [50,51]. The corrosion resistance of Inconel 718 fabricated by cold metal transfer-based wire arc additive manufacturing (CMT-WAAM) was improved using post-heat treatment [52]. The authors applied a multi-step heat treatment including homogenization, solution treatment, and aging. It resulted in, inter alia, reduced porosity, and more uniformly distributed alloy components. Solution treatment in a vacuum furnace is also a promising technique to improve the corrosion-wear resistance of SLM Inconel 718 [53]. Moreover, 3D prints after hot isostatic pressing shows reduced level of porosity and the absence of the dendritic structure, which also results in an improvement of corrosion resistance [54].

In this work, the corrosion resistance of Inconel 718 produced by the LPBF method was evaluated in a 3.5% wt. NaCl solution. Despite extensive research on the corrosion behavior of Inconel 718 produced by laser powder bed fusion, most studies have focused on classical process parameters such as laser power, scanning speed, hatch spacing, linear energy density, or post-processing heat treatments. Although the influence of build orientation is occasionally reported, it is typically limited to evaluating specimens printed strictly in vertical or horizontal orientations, and thus treats orientation only as a general manifestation of manufacturing-induced anisotropy. Consequently, the literature does not address the inclination angle of the printed surface as a controlled and independent structural parameter.

In PBF-LB/M, the local angle of the surface relative to the laser beam directly determines melt-pool behavior, thermal gradients, layer–layer overlap, and the magnitude of the staircase effect. These factors strongly affect surface morphology, distribution of microstructural defects, and the exposure of specific crystallographic domains. However, the relationship between inclination angle and corrosion susceptibility has been largely overlooked. The limited references that mention orientation effects do not perform a systematic, multi-angle analysis, nor do they correlate the angle-dependent surface and microstructural features with electrochemical kinetics or elemental dissolution behavior.

For materials such as Inconel 718, which rely on passive-film stability to ensure long-term performance in chloride-containing environments, even subtle variations in surface morphology, grain orientation, or segregation of alloying elements can significantly alter corrosion mechanisms. Understanding how these features evolve with surface inclination is therefore crucial for optimizing the design of additively manufactured components, particularly those exposed to aggressive media in aerospace, energy, or marine applications.

The present study addresses this knowledge gap by conducting a comprehensive, multi-method investigation of the effect of inclination angle on the corrosion resistance of PBF-LB/M Inconel 718. By examining long-term immersion behavior, potentiodynamic polarization response, surface morphology evolution (SEM/EDS), roughness development, and elemental dissolution (MP-AES), this work demonstrates that inclination angle is not merely a geometric attribute but a key structural factor governing corrosion kinetics. The results reveal measurable and systematic differences in Icorr values, mass loss, passive layer stability, and degradation morphology across various inclination angles.

## 2. Materials and Methods

### 2.1. Sample Fabrication and Preparation

The test samples were produced by the PBF-LB/M method from IN718 nickel alloy powder (NiCr19Fe19Nb5Mo3, 2.4668, Table 1), supplied by Rosswag GmbH (Pfinztal, Germany).

The chemical composition of the powder (Table 2) was analyzed using the wavelength-dispersive X-ray fluorescence (WDXRF) method with a Rigaku Primini spectrofluorometer (Rigaku, Tokyo, Japan).

The results of the performed analysis show that the contents of Al, Co, Cr, Fe, and Ni are within the range declared by the producer, while those of Mo and Nb are slightly higher. Ti, not mentioned by the producer, was detected.

The inclined elements were 3D printed using an XM200C printer (Xact Metal, State College, PA, USA). Process parameters such as layer thickness, hatch distance, scanning strategy, laser spot diameter, and laser power and speed for the bottom, middle, and top layers are presented in Table 3. These values ensured stable melting conditions and uniform material consolidation throughout the structure. The printing process was conducted in a protective atmosphere of argon, with parameters ensuring complete powder melting and a stable molten metal pool.

**Figure 1:**
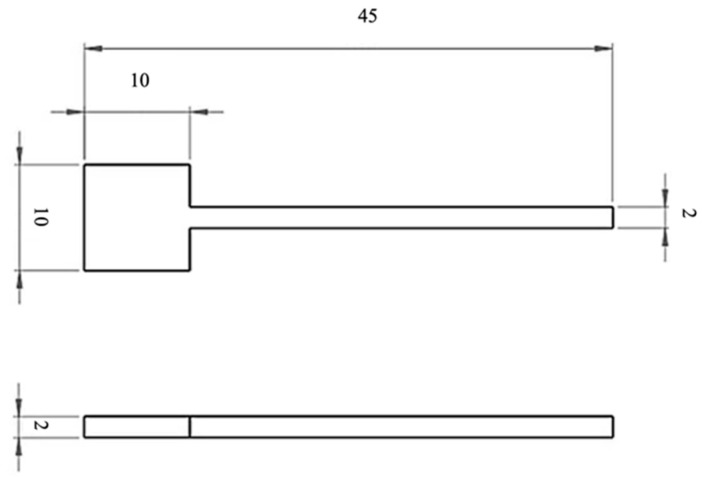
Dimensions of samples printed using PBF-LB/M technology.

The samples were manufactured according to the dimensions shown in Figure 1.

**Figure 2:**
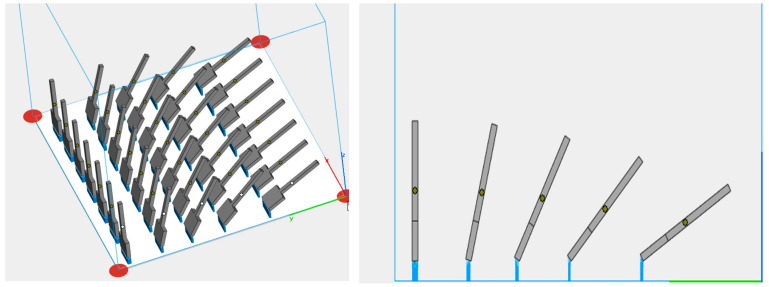
Samples orientation on the build platform in Materialize Magics—0°, 15°, 30°, 45°, and 60°.

Each element was printed tilted relative to the vertical by the following angles: 0°, 15°, 30°, 45°, and 60° (Figure 2).

After the printing process was completed, all samples were cut from the build platform using the electro-discharge method. After removal from the build platform, all specimens were subjected to ultrasonic cleaning in distilled water for 20 min to remove loosely attached, non-fused powder particles. Following cleaning, the samples were visually inspected to ensure the absence of visible residual powder prior to corrosion testing. The printed components were not subjected to any finishing or mechanical processing, which allowed for the preservation of the surface condition characteristic of the PBF-LB/M technology and a clear assessment of the effect of post-print topography on corrosion behavior. Rolled sheets of Inconel 718 alloy were used as a reference sample. These samples constituted a reference material enabling comparison of the corrosion behavior and electrochemical properties of the alloy obtained using the conventional and additive methods. The rolled sheets were subjected to an identical range of tests, including immersion tests, electrochemical measurements, and topography and microstructure analysis.

### 2.2. Long-Term Immersion Corrosion Testing

The alloy’s corrosion resistance was assessed based on a long-term immersion test lasting 1000 h, conducted in a 3.5% aqueous NaCl solution (POCH, p.a., Gliwice, Poland). The experiment was conducted simultaneously at two temperature conditions: room temperature (20 °C) and elevated temperature (45 °C). The solutions were not stirred during the corrosion process at both temperatures. Sample mass measurements, necessary to determine material loss, were performed using a Radwag AS 60/220 R2 analytical balance (RADWAG, Radom, Poland) with an accuracy of 0.00001 g. The immersed surface areas were equal for the 3D prints and the foil. The corrosion rate *Vc* was calculated using the following formula:

$$Vc=\frac{∆m}{S\cdot t}\;[g/{cm}^{2}\cdot year]$$

where

- *Vc* is the corrosion rate, defined as the loss of one gram of metal per square centimeter of surface area per year;
- Δ*m* is the difference in sample mass before and after the corrosion test (g);
- *S* is the sample area (cm^2^);
- *t* is the duration of the corrosion test (year).

The corrosion mechanism of IN718 alloy in a chloride environment is electrochemical in nature, where microstructural heterogeneity resulting from 3D printing of elements tilted at various angles promotes the formation of local anodic and cathodic cells. This process involves an anodic reaction involving the oxidation of nickel as the main component of the matrix:

(1) $$Ni\to {Ni}^{2+}+2\bar{e}$$

At the same time, a cathodic reaction occurs, consisting of the reduction of oxygen in the presence of water, which leads to the formation of hydroxide ions:

(2) $$O_{2}+2H_{2}O+4e^{-}\to 4{OH}^{-}$$

Corrosion products are formed as a result of secondary reactions between the resulting ions:

(3) $${Ni}^{2+}+2{OH}^{-}\to \;{Ni(OH)}_{2}$$

### 2.3. Potentiodynamic Polarization Measurements

Potentiodynamic polarization measurements were performed on independent, freshly prepared specimens that had not been previously subjected to immersion corrosion testing or any other electrochemical measurements. This approach was adopted to eliminate any influence of surface pre-degradation on electrochemical parameters. Potentiodynamic polarization tests were performed on foil and 3D prints with different inclination angles using BioLogic SP-200 potentiostat (Seyssinet-Pariset, France). The polarization curves were measured potentiodynamically by linearly sweeping the potential from −2 V to +2 V relative to the open-circuit potential (OCP) at a rate of 0.01 V/s. The measurement was conducted in a vessel containing 3.5% NaCl solution with a saturated calomel electrode (SCE) serving as the reference electrode, a platinum plate at least ten times larger than the surface of the test samples serving as the counter electrode, and the test samples serving as the working electrode.

### 2.4. Surface and Chemical Characterization

**Figure 3:**
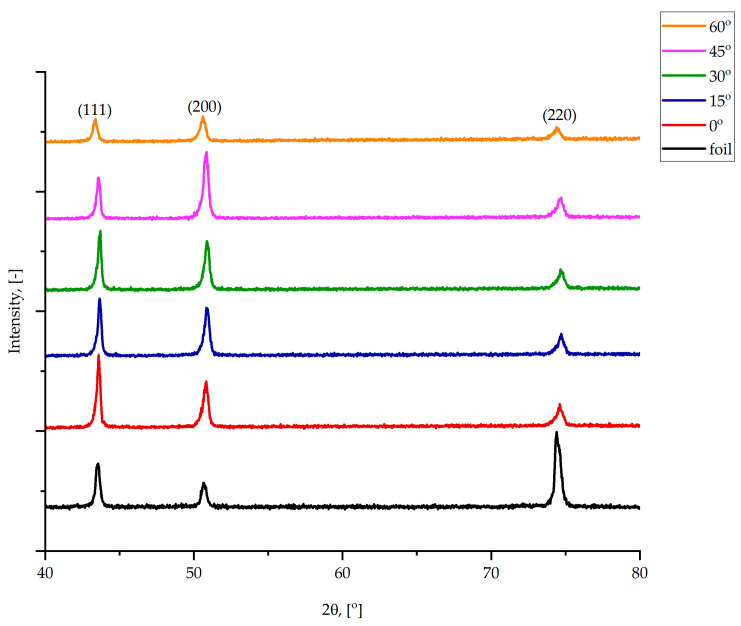
X-ray diffraction patterns.

Before corrosion testing, X-ray diffraction (XRD) analysis was performed on all samples to identify and compare the phases present in the material produced by conventional and additive methods. The phase composition of Inconel alloy 718 was analyzed using an X-ray Diffraction (XRD) Spectrometer (Rigaku Miniflex II, Rigaku, Tokyo, Japan) equipped with a copper tube. Scans were performed from 40° to 80° at a scan speed of 1°/min. Possible differences in phase composition between samples were investigated using the XRD technique (Figure 3).

Obtained diffraction patterns revealed that there is no difference in phase composition between Inconel alloy 718 in the form of foil and 3D prints. Detected peaks are associated with the face-centered cubic (FCC) γ-phase, and they are already observed in IN718 samples fabricated using the PBF-LB/M method [56]. Significantly higher peak (220) for foil IN718 contributes to the direction of rolling. A slight influence of the applied angle of inclination on the intensity of (111) and (200) peaks is noticeable. No peaks from Laves phases at 2ϑ = 47.82° and 77.56° were observed [57]. Crystalline grain sizes were determined from X-ray diffraction patterns using the (111) peak via the Scherrer equation. Results are listed in Table 4.

Changing the angle of inclination from 0° to 45° does not influence crystallite sizes. However, for the sample printed with an angle of 60°, crystalline grain sizes are smaller and similar to those of foil IN718. A smaller crystallite size means a higher boundary area, which can act as a corrosion center.

Surface topography and roughness parameters Ra and Rz were examined using a Veeco Wyko NT9300 optical profilometer (Veeco, United States). Measurements were taken at three points for all samples, before and after the corrosion process. The research was supplemented by microstructural analysis using a JEOL JCM-6000 PLUS scanning electron microscope (SEM) (JEOL, Tokyo, Japan) equipped with an Energy Dispersive X-ray Spectrometer (EDS) JED-2300, used to analyze the chemical compositions of samples and corrosion products. Macroscopic images of the samples before and after corrosion testing were acquired using a Nikon D850 digital single-lens reflex (DSLR) camera (Nikon Corporation, Tokyo, Japan) under controlled lighting conditions and fixed focal distance to ensure consistent and reproducible documentation. To control and quantify the dissolution of alloy components during the measurements, a fixed volume of solution was withdrawn after 1000 h of corrosion tests at 20 °C and 45 °C and analyzed using the MP-AES technique (Microwave Plasma–Atomic Emission Spectroscopy, Agilent 4200, Agilent Technologies, Santa Clara, CA, USA). Before sample analysis, the instrument was calibrated for all investigated metals, including Ni, Fe, Ti, Al, Co, Cr, Mo, Mn, and Nb. Calibration solutions were prepared at concentrations of 0 ppm, 0.5 ppm, 1 ppm, 5 ppm, 10 ppm, and 20 ppm by diluting certified 1000 ppm single-element standards (PlasmaCAL, Villebon-sur-Yvette, France) with 1 M nitric acid (POCH, p.a., Gliwice, Poland). Each standard was analyzed under identical operating conditions, and the measured intensities were used to construct calibration curves. For all elements, the linear regression coefficient of the calibration curves (R^2^) was at least 0.9995, confirming linearity of detector response within the selected working range. It is essential to ensure that the analyte concentrations in the post-corrosion test solutions fall within the range defined by the calibration standards. If the measured concentration exceeded this range, the solution was diluted with 1 M nitric acid, accordingly, before analysis. In the present study, selected post-corrosion solutions required a ten-fold dilution, which was taken into account by the instrument software during data acquisition and quantitative evaluation.

## 3. Results and Discussion

### 3.1. Long-Term Immersion Corrosion Testing

Macroscopic analysis of the sample surface before and after the long-term corrosion test showed the influence of both the environmental temperature and the angle of inclination of the samples in the PBF-LB/M process on the nature and intensity of surface degradation (Table 5).

Samples subjected to long-term corrosion at 20 °C showed no significant visual changes. Changes in surface degradation were observed after exposure at 45 °C. In this case, local areas of intense corrosion were visible, especially for samples printed at higher inclination angles. This indicates a significant effect of temperature on the kinetics of electrochemical processes and the stability of the passive layer on the Inconel 718 alloy surface. In the case of the reference sample, rolled sheet metal, no significant macroscopic changes were observed after exposure at 20 °C and 45 °C. The surface remained homogeneous, with no visible signs of local degradation.

**Figure 4:**
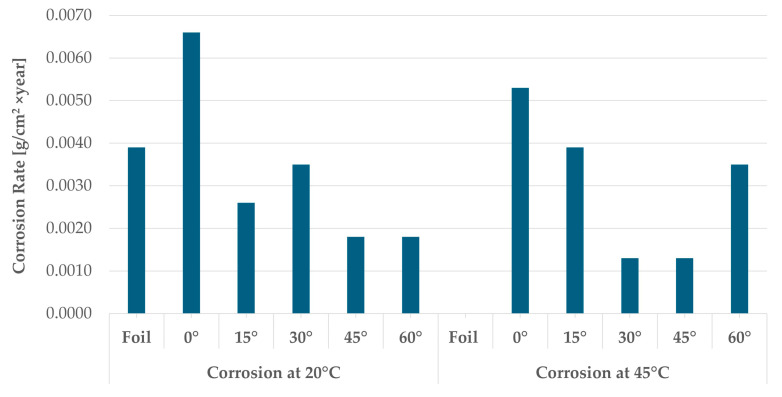
Relationship between corrosion rate, sample inclination angle, and corrosion temperature.

Samples before and after the corrosion test were weighed to determine the Average Mass Loss and Average Corrosion Rate (Table 6, Figure 4).

It can be concluded that the highest corrosion rate is shown by the sample printed at an inclination angle of 0°, the remaining 3D-printed samples have these values comparable to or lower than the foil. At 45 °C, it is the opposite. There is no corrosion of the foil observed. In the case of printed components, the corrosion rate is significantly lower at 30° and 45° compared to the values at 20 °C. There is no clear dependency between the temperature of the environment and the corrosion rate of the samples. It is related to the rate of dissolution of corrosion products from the surface, and thus the mass loss depends on the temperature, the angle of inclination, and, therefore, the development of the surface.

### 3.2. Potentiodynamic Polarization Measurements

**Figure 5:**
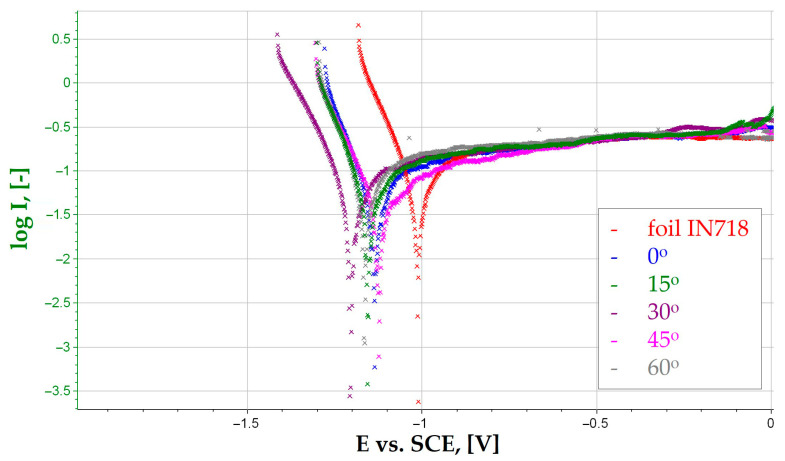
Potentiodynamic test graph for foil IN718 and printed samples with different angles of inclination.

The polarization curves obtained for IN718 alloy samples, both produced by the PBF-LB/M method at different inclination angles, are presented in Figure 5.

Analysis of the polarization curve shapes indicates the presence of distinct active solubility regions and a tendency toward limited behavior in the anodic potential range. All samples exhibit a lack of a stable passive range, which is typical for nickel alloys exposed to chloride environments, where Cl^−^ ions destabilize the passive layer and promote local pitting corrosion processes. Samples printed at higher inclination angles (45° and 60°) exhibit greater current fluctuations in the anodic range, which can be attributed to local breakdown of the passive layer and the initiation of local corrosion processes in areas of increased roughness and heterogeneous chemical composition. Comparison of the additive samples with the rolled sample clearly indicates that the manufacturing technology is crucial for the electrochemical behavior of the IN718 alloy. 3D printing using the PBF-LB/M method creates microstructures with increased anisotropy and heterogeneity, which is directly reflected in shifts in corrosion potential and changes in the intensity of corrosion currents. The results also show that the appropriate selection of printing orientation can limit the rate of corrosion processes, which has significant application implications in the context of designing components operating in aggressive environments. The curves presented in Figure 5 provide the electrochemical parameter values Ecorr and Icorr (Table 7). The corrosion potential (Ecorr) represents the thermodynamic equilibrium of the system at which the rates of anodic and cathodic reactions are equal, while the corrosion current density (Icorr) serves as a kinetic parameter directly proportional to the material’s degradation rate. These electrochemical parameters were determined by Tafel extrapolation of the polarization curves to assess the corrosion resistance of the studied samples.

Corrosion potential (Ecorr) is a measure of the thermodynamic tendency of a material to enter an active state in a given electrolytic environment. The highest value was recorded for the rolled sample (−1019 mV), indicating its relatively more noble character compared to the additive samples. This is a consequence of a more uniform microstructure and fewer surface and volumetric defects typical of materials obtained by conventional methods. Samples printed using the PBF-LB/M method are characterized by a shift in the Ecorr parameter towards more negative values, with the range being from −1124 mV (45°) to −1203 mV (30°). The most negative corrosion potential obtained for the sample printed at an angle of 30° indicates its highest thermodynamic susceptibility to the initiation of corrosion processes. This phenomenon can be attributed to local microstructural heterogeneity and an increased number of topographic discontinuities, which favor the formation of electrochemical microcells. The lack of a monotonic dependence on the inclination angle suggests that the electrochemical character of the surface is determined by a complex combination of factors, such as the geometry of the liquid metal pool, the direction of crystallization, local roughness, and the distribution of residual stresses, rather than solely by the sample orientation angle. Samples characterized by higher Icorr values (rolled sheet and the sample printed at an angle of 30°) simultaneously exhibited increased mass loss after 1000 h of exposure in NaCl solution, confirming that the intensification of anodic reactions leads to faster dissolution of the nickel matrix. This consistency confirms the correctness of the electrochemical prediction of corrosion rate based on parameters determined from polarization curves. In turn, the sample printed at 0°, which had the lowest Icorr value, also exhibited the lowest corrosion rate, *Vc*, which clearly indicates the more favorable corrosion resistance of this orientation variant. This means that in this case, anodic processes occur more slowly, and material degradation is more limited.

Samples printed using the PBF-LB/M method are characterized by a shift in the Ecorr parameter towards more negative values, with the range being from −1124 mV (45°) to −1203 mV (30°). The most negative corrosion potential obtained for the sample printed at an angle of 30° indicates its highest thermodynamic susceptibility to the initiation of corrosion processes. This phenomenon can be attributed to local microstructural heterogeneity and an increased number of topographic discontinuities, which favor the formation of electrochemical microcells. The lack of a monotonic dependence on the inclination angle suggests that the electrochemical character of the surface is determined by a complex combination of factors, such as the geometry of the liquid metal pool, the direction of crystallization, local roughness, and the distribution of residual stresses, rather than solely by the sample orientation angle. Samples characterized by higher Icorr values (rolled sheet and the sample printed at an angle of 30°) simultaneously exhibited increased mass loss after 1000 h of exposure in NaCl solution, confirming that the intensification of anodic reactions leads to faster dissolution of the nickel matrix. This consistency confirms the correctness of the electrochemical prediction of corrosion rate based on parameters determined from polarization curves. In turn, the sample printed at 0°, which had the lowest Icorr value, also exhibited the lowest corrosion rate, *Vc*, which clearly indicates the more favorable corrosion resistance of this orientation variant. This means that in this case, anodic processes occur more slowly and material degradation is more limited.

Corrosion current (Icorr) is a kinetic parameter directly related to the rate of electrochemical reactions and the intensity of material degradation. The lowest value (29 µA) was recorded for the sample printed at an angle of 0°, indicating the lowest rate of corrosion processes in this group of additive samples. This phenomenon can be interpreted as a result of more uniform melting of subsequent layers and a more favorable alignment of the scanning paths relative to the surface exposed to the electrolyte. The highest Icorr values were obtained for the rolled sample (57 µA) and the sample printed at an angle of 30° (59 µA). In the case of the rolled material, the increased corrosion current, despite the more positive Ecorr, may result from the nature of the passive layer, which is locally weakened in a chloride environment, leading to an increase in the intensity of anodic reactions. For the 30° sample, the high Icorr value indicates that surface and microstructural defects have a dominant influence, increasing the effective surface area for electrochemical reactions. The remaining additive samples (15°, 45°, and 60°) exhibit intermediate corrosion current values, suggesting moderate kinetics of corrosion processes and confirming the significant effect of print orientation on the intensity of electrochemical degradation. The corrosion potential Ecorr does not show a clear quantitative correlation with the corrosion rate. Ecorr reflects the thermodynamic tendency to initiate corrosion, while Icorr determines the kinetics of the process and thus the actual rate of material loss.

### 3.3. Surface and Chemical Characterization

The morphology of the samples was analyzed before and after the corrosion test. Images are presented in Table 8.

SEM images taken after the corrosion test revealed distinct differences in the nature of surface degradation depending on temperature and printing orientation. For the rolled sample, the SEM images did not reveal any significant pitting or extensive material loss after both 20 °C and 45 °C testing. At 20 °C, the 3D-printed samples exhibited surface changes, primarily involving the removal of loosely bound powder particles and rounding of sharp irregularities. No extensive areas of pitting corrosion were observed, confirming the stable nature of passivation under these conditions. At 45 °C, SEM images revealed local areas of intense degradation, particularly visible for the 45° and 60° inclination angles. In these areas, structures characteristic of localized corrosion were observed, including pitting and irregular material loss.

Chemical composition analyses were also performed using the EDS method (Table 9).

EDS analysis of the sample surfaces before and after the corrosion test revealed clear changes in chemical composition resulting from the chloride environment and exposure temperature. Before the corrosion process, the oxygen content on the surface of all samples was similar, indicating a comparable state of the passive layer regardless of the manufacturing technology and inclination angle.

The main alloy component, nickel (Ni), exhibited a stable level of 41.7–42.6 atomic percent for all variants before corrosion. After 1000 h of exposure in 3.5% NaCl, the nickel content on the surface fluctuated slightly; at 45 °C, a decrease was observed for samples with high inclination angles (45° and 60°), suggesting more intense Ni^2+^ ion transfer into solution in areas of high roughness.

Chromium (Cr), responsible for passivation, exhibited a concentration of approximately 18.0–19.6 at.% before the test. for 3D prints and significantly lower (13.2 at.%) for the reference foil. After testing at 20 °C, the chromium content remains stable, confirming the durability of the passive layer. However, at 45 °C, a local decrease in chromium was observed for the 60 °C sample, which coincides with the occurrence of pitting corrosion. Iron (Fe) remains at 14.3–16.6 at.%, demonstrating the greatest stability in the foil and the 0° print, while in the 45° and 60° samples, its content dissipates after testing at 45 °C.

Niobium (Nb) and molybdenum (Mo) are key to local corrosion resistance. Niobium in the prints fluctuates around 2.7–3.0 at.%, and molybdenum around 1.4–1.6 at.%. It is worth noting that the foil has a lower niobium concentration (1.66 at.%) than the prints, which is due to differences in the technological process (rolling vs. PBF-LB/M). After corrosion at 45 °C, samples with 45° and 60° angles showed increased niobium concentrations in micro-regions, which may indicate selective dissolution of the nickel matrix and exposure of Nb-rich phases. Titanium (Ti) and aluminum (Al) are present in smaller amounts (Ti approximately 1.2 at.%, Al approximately 1.9–3.0 at.% in the prints). An increase in temperature to 45 °C did not cause rapid leaching of these elements, indicating their stable anchoring in the structure of the solid solution or precipitates. Carbon (C) shows the highest concentrations in the foil (18.3 at.%) and the 60° sample (13.3 at.%), which may be related to surface contamination or the presence of carbides, which, in the case of the 60° sample, may constitute additional corrosion initiation sites due to the high roughness of this surface.

**Figure 6:**
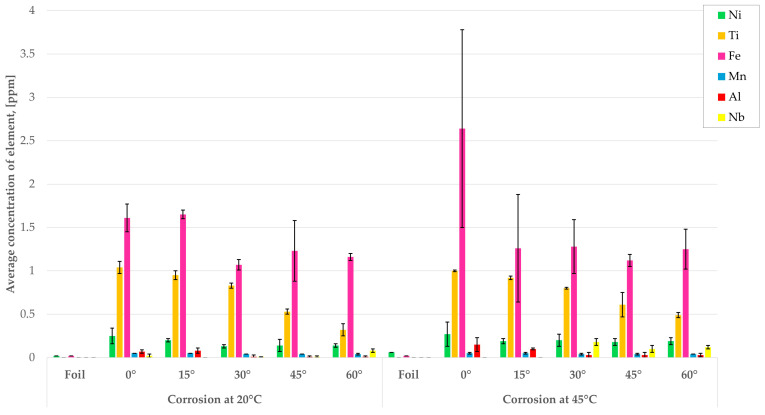
Average concentration of components of alloy in the solutions after corrosion.

To control the dissolution of components of IN718, post-corrosion solutions were analyzed using the MP-AES technique. Results are summarized in Figure 6.

Nickel is present in all post-corrosion solutions, with the highest concentration observed after tests of the sample printed at a 0° angle of inclination. No strong effect of applied temperature on its dissolution is observed. At the same time, the clear dependence between the angle of inclination and the average concentration of the element dissolved can be observed for Ti. Its amount decreases with the increase in the inclination angle. However, the application of higher temperature (45 °C) does not enhance its dissolution. The corrosion rate of Fe is the highest among all metals, especially at 0 °C and 45 °C. Manganese is always present in the post-corrosion solution, but its concentration is constant and irrelevant to applied angles and temperatures. Higher concentrations of Al are observed for samples printed with angles of 0° and 15°, but with no significant temperature effect. The largest amount of Nb dissolved was for the sample printed with 60° of inclination, with the enhancement in its corrosion rate at higher temperatures. Co, Cr, and Mo were not detected in the analyzed solutions. It can be concluded that Ni, Ti, and Fe are the main metals dissolving in 3.5% NaCl. However, applied temperature does not strongly affect the corrosion rate of IN718. Besides Nb, with lower values of inclination angles (mostly 0° and 15°), the concentration of elements in post-corrosion solutions. In the case of Inconel alloy 718 foil, only low concentrations of Ni and Fe were detected in the solutions. It means that the alloy in the form of 3D prints shows worse corrosion resistance than the bulk material.

The results of the analysis of the chemical composition of the post-reaction solutions closely correlate with the results of the EDS analysis of the sample surfaces. Increased concentrations of nickel, iron, and titanium in the solutions after the corrosion test, particularly at 45 °C, correspond to a decrease in the relative share of these elements in the material’s surface layer, confirming their selective dissolution during exposure to a chloride environment. For samples printed at higher inclination angles, higher concentrations of alloying elements in the solution and increased oxygen content were observed in EDS analyses, indicating the coexistence of two parallel mechanisms: metal dissolution and the accumulation of corrosion products on the material surface. This degradation pattern explains the apparent weight increase in the samples observed in the gravimetric tests. In turn, the lower concentrations of elements in the solutions for the rolled sheet and the smaller changes in surface composition confirm the greater stability of the passive layer of the material produced by the conventional method. The obtained results clearly indicate that the analysis of post-reaction solutions should be interpreted together with the EDS results to better understand the corrosion mechanism of nickel alloys under complex operating conditions.

**Figure 7:**
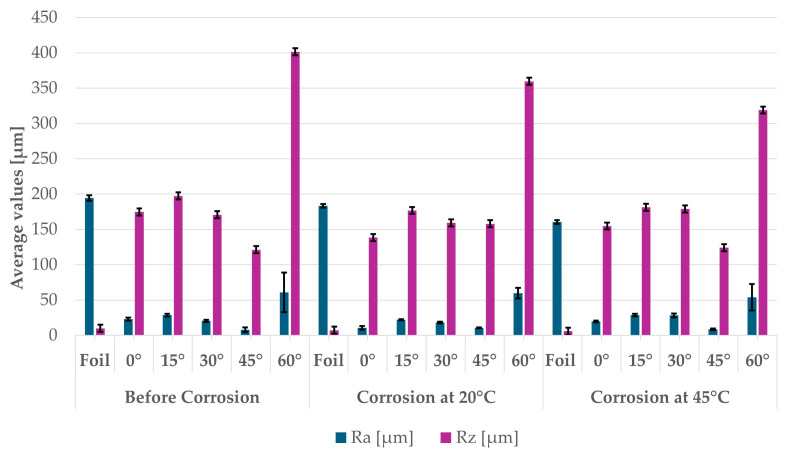
Graph of average Ra and Rz values for tested surfaces depending on the inclination angle in the additive manufacturing process and the temperature of the corrosive environment.

The surface topography of the samples was analyzed before and after the long-term corrosion exposure process (Table 10, Figure 7).

The observed changes are a direct result of the synergistic interaction of three key factors: the temperature of the corrosive environment, the anisotropic surface topography resulting from the PBF-LB/M technology specifications, and the characteristic microstructure of the Inconel 718 alloy immediately after printing. Unlike conventionally manufactured materials, PBF-LB/M printed parts are characterized by strong near-surface heterogeneity, the nature of which is largely determined by the element’s inclination angle relative to the build platform. A pronounced roughness anisotropy, characteristic of additive technologies, is also observed, resulting from the layered nature of the process, the stepping effect, the balling effect, and local disturbances in the stability of the liquid metal pool. Importantly, this type of topography is not merely a geometric feature but directly influences the corrosion behavior of the surface. Surfaces with high initial roughness are characterized by an increased effective contact surface with the electrolyte and numerous micro-cavities that promote local retention of the corrosive solution, leading to the initiation of local corrosion processes. The presence of residual stresses causes local variations in corrosion resistance in the subsurface layer.

The sample after the rolling process was characterized by significantly lower Rz values and a more uniform surface profile compared to the additive samples. At both 20 °C and 45 °C, changes in the Ra and Rz parameters were gentle, indicating gradual surface smoothing rather than the development of local degradation. This behavior confirms that low initial roughness limits the possibility of initiating corrosion processes and promotes the stability of the passive layer. Analysis of the initial state of samples produced by the PBF-LB/M method revealed a clear and significant correlation between the angle of geometric inclination relative to the build platform and the quality of the surface topography. Samples inclined at an angle of 45° had the lowest initial roughness, for which the mean values of the Ra and Rz parameters were 8.05 µm and 121.28 µm, respectively. This orientation favored obtaining a relatively homogeneous surface, which can be attributed to the reduced stepping effect and a more stable flow of the liquid metal pool during the PBF-LB/M process. As the inclination angle increased to 60°, a rapid degradation of the surface geometric parameters was noted, manifested by an increase in Ra to 60.85 µm and Rz to 401.39 µm. This phenomenon is associated with surface formation mechanisms typical of selective laser melting of powders, such as intense adhesion of incompletely melted powder particles, the presence of depressions, and local subsurface discontinuities.

Corrosion testing conducted at 20 °C for 1000 h resulted in significant changes in the surface morphology of the tested samples, with the nature of these changes closely dependent on the initial topography resulting from the print orientation. For samples printed at 0° and 30° angles, corrosion primarily affected the most protruding and energetically unfavorable surface areas. Sharp irregularities and loosely bound powder particles, which remain on the surface after the additive manufacturing process, were primarily removed. As a result, a significant reduction in surface roughness was noted, with the Ra parameter decreasing by over 50% for the 0° orientation. The impact of the corrosive environment at room temperature was moderate and did not lead to significant surface degradation. This effect can be attributed to the formation of a stable passive layer, which provided effective electrochemical protection and limited the development of pit corrosion. Under these conditions, material loss was concentrated primarily on the most prominent topographic features, leading to partial surface smoothing. A different behavior was observed for samples with high initial roughness, especially for the 60° orientation. High surface inhomogeneity, the presence of numerous microcavities, and adhering powder particles favored local electrolyte retention. This phenomenon limited the effectiveness of the smoothing process, resulting in very high Ra and Rz parameter values, even after 1000 h of exposure at 20 °C. These results indicate that, in the case of high initial roughness, the protection provided by the passive layer is not sufficient to significantly improve the quality of the surface topography.

A significant change in the surface degradation pattern was observed after increasing the corrosive environment temperature to 45 °C. The elevated temperature weakened the stability of the passive layer and accelerated ion transport in the near-surface layer. Under these conditions, corrosion ceased to be selective and began to develop locally. This phenomenon was particularly noticeable on surfaces containing numerous irregularities and geometric defects, typical of components printed at higher inclination angles, especially 45° and 60°. In the case of samples printed at an angle of 60°, the highly developed surface topography favored local differences in corrosion conditions. Combined with the elevated temperature, this led to a loss of the protective properties of the passive layer and the development of pitting corrosion. The result of these processes was an increase in the Ra and Rz parameters, indicating a further increase in surface irregularities and the formation of deep material defects. An additional factor influencing the degradation process was the heterogeneity of the near-surface layer, characteristic of components manufactured using the PBF-LB/M method, which favored localized corrosion, especially at elevated temperatures. Samples printed at an angle of 45°, characterized by a more uniform surface and fewer defects, exhibited fewer corrosion initiation sites. As a result, even at 45 °C, the increase in roughness for this orientation was limited. Images of surface topography for all samples are shown in Table 11.

The obtained results show that the corrosion resistance of Inconel 718 additively manufactured components depends on the print orientation and corrosion temperature. The 45° orientation provided the highest topography stability within the analyzed temperature range, making it the most favorable for the durability of components operating in corrosive environments. At the same time, increasing the temperature significantly increased the material’s sensitivity to surface defects, making samples with high initial roughness, especially those printed at a 60° angle, the most susceptible to accelerated corrosion.

Analysis of the gravimetric test results allowed for a preliminary assessment of the intensity of corrosion processes depending on the sample inclination angle and ambient temperature. At 20 °C, printed samples, besides one with an inclination angle of 0°, exhibited small mass losses, but the lowest values were recorded for samples produced at a 45° angle, indicating their more favorable corrosion behavior at room temperature. At 45 °C, the gravimetric method revealed significant differences between the print orientations. However, analysis of the results in comparison to other test techniques indicates that samples with a 45° inclination angle exhibited a more stable corrosion process than samples printed at a 60° angle.

The electrochemical test results confirmed the significant effect of inclination angle on the kinetics of corrosion processes. At 20 °C, the lowest corrosion current densities and the most favorable electrochemical parameter values were obtained for samples produced at a 45° angle, indicating greater stability of the passive layer. At 45 °C, a general increase in electrochemical activity was observed, but samples printed at a 45° angle still exhibited a more controlled corrosion process compared to samples with a higher inclination angle.

SEM observations revealed distinct differences in the surface morphology of the samples after the corrosion test. At room temperature, the surface of samples printed at a 45° angle was relatively uniform, with a limited number of local degradation points. At 45 °C, the intensity of morphological changes increased, but samples printed at a 45° angle exhibited less advanced surface degradation compared to samples printed at a 60° angle, for which more developed corrosion product structures were observed.

EDS analysis provided significant information regarding the behavior of individual alloying elements during the corrosion process. Changes in nickel content indicate its active participation in the dissolution processes, particularly at 45 °C, with the smallest changes in Ni concentration observed for samples printed at a 45° angle. Chromium, the key element responsible for the alloy’s passivation, exhibited greater stability at 20 °C, while at elevated temperatures, partial surface depletion was observed, more pronounced for samples printed at 60°.

Iron tended to selectively dissolve at 45 °C, as evidenced by changes in its percentage on the surface of samples printed at higher inclination angles. Titanium and niobium, present in the alloy’s strengthening phases, exhibited less pronounced concentration changes, but their relative depletion in the 60° samples suggests local microstructural destabilization under more intense corrosion conditions.

Overall, EDS analysis indicates that samples printed at 45° had the most stable chemical composition of the surface layer at both 20 °C and 45 °C. The results of the gravimetric, electrochemical, microstructural, and EDS analyses clearly indicate that, among the additively printed samples analyzed, the best corrosion properties at 20 °C were exhibited by those produced at a 45° inclination angle. Also, at an elevated temperature of 45 °C, this print orientation provided the most stable corrosion behavior, with limited surface degradation and smaller changes in chemical composition compared to the other samples.

## 4. Conclusions

The obtained results led to the following conclusions:

- The conducted studies clearly confirm that the corrosion resistance of Inconel 718 alloy in chloride environments is related to the material’s chemical composition, manufacturing technology, surface topography, and the diversity of the near-surface layer. Despite its numerous design advantages, the PBF-LB/M technology leads to the formation of an anisotropic microstructure and a highly diversified surface topography, which significantly modify the mechanisms of initiation and development of corrosion processes.
- It was demonstrated that print orientation is crucial for the electrochemical stability of the surface. No monotonic relationship was observed between the sample tilt angle and the corrosion rate, indicating that corrosion resistance is determined by a complex combination of factors, such as the nature of the topography, local crystallization conditions, and the distribution of defects. Of the variants analyzed, the 45° orientation provided the most balanced surface condition, combining relatively low roughness with a limited number of local corrosion initiation sites, which translated into the highest stability under the tested environmental conditions.
- Increasing the temperature of the corrosive environment did not alter the material’s degradation mechanism. At 20 °C, selective surface smoothing processes dominated, consisting primarily of the removal of energetically unfavorable topographic features and loosely bound powder particles. Under these conditions, the passive layer maintained relative stability, effectively limiting the development of local corrosion. However, at 45 °C, a significant weakening of the corrosion rates of the foil and samples printed with 30° and 45° angles was observed. Due to the rate of dissolution of corrosion products from the surface, the mass loss depends on the development of the surface. The corrosion resistance of components cannot be simply explained only by mass loss measurements.
- Comparison of the additive samples with the reference sample of rolled sheet metal revealed fundamental differences in the nature of corrosion behavior. The uniform microstructure and low surface roughness of the sheet metal favored the formation of a more stable passive layer, which limited the number of active electrochemical reaction sites. At the same time, the electrochemical results indicate that even conventional materials can exhibit increased anodic kinetics following local damage to the passive layer, highlighting the significant role of environmental conditions in determining the actual corrosion resistance of the alloy.
- Analysis of the correlation between electrochemical parameters, mass loss, and changes in surface topography confirmed that the actual degradation rate is primarily determined by kinetic parameters and local near-surface conditions, which, in the case of components manufactured using the PBF-LB/M method, are strongly dependent on the print orientation and surface quality immediately after production.
- A comparative analysis of all the tests performed showed that among the additively printed samples, the 45° orientation provided the best corrosion resistance both at room temperature (20 °C) and at elevated temperature (45 °C), regardless of the evaluation method used.
- The obtained results have significant application and design implications. They indicate that the corrosion resistance of additively manufactured components can be significantly influenced at the design stage of the printing process through the appropriate selection of geometric orientation. This can significantly improve the corrosion resistance of Inconel 718 alloy components. This is of key application importance in the context of designing components intended for use in aggressive environments, especially at elevated temperatures.

## Figures and Tables

**Table 1:** Chemical composition of IN718 powder used to produce samples using the PBF-LB/M method [55].

	Chemical Composition of the IN718 Powder									
Element	Al	C	Co	Cr	Cu	Fe	Mn	Mo	Nb + Ta	Ni
Mass percentage [%]	0.20 ÷ 0.80	≤0.08	≤1.00	17.00 ÷ 21.00	≤0.30	Balance	≤0.35	2.80 ÷ 3.30	4.75 ÷ 5.50	50.00 ÷ 55.00

**Table 2:** Chemical composition of IN718 powder determined using the WDXRF method.

	Determined Chemical Composition of the IN718 Powder							
Element	Al	Ti	Co	Cr	Fe	Mo	Nb	Ni
Mass percentage [%]	0.2142	0.8529	0.0631	19.0345	17.5459	3.6071	5.7444	52.9379

**Table 3:** PBF-LB/M printing process parameters for IN718.

Parameter	Value
Layer thickness	30 µm
Hatch distance	0.08 mm
Scanning strategy	Alternate scanning, 67° rotation per layer
**Down skin**	
Laser power	40 W
Scanning speed	500 mm/s
Laser spot diameter	0.1 mm
**In skin**	
Laser power	70 W
Scanning speed	500 mm/s
Laser spot diameter	0.1 mm
**Up skin**	
Laser power	80 W
Scanning speed	550 mm/s
Laser spot diameter	0.1 mm

**Table 4:** Crystallite sizes calculated using the Scherrer equation.

Angle of Inclination	Crystallite Sizes [nm]
foil	32
0°	49
15°	47
30°	47
45°	48
60°	28

**Table 5:** Results of macroscopic surface analysis before and after long-term corrosion testing.

Sample	Before Long-Term Corrosion Test	After Long-Term Corrosion Test	
		20 °C	45 °C
Foil	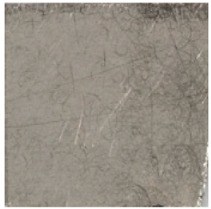	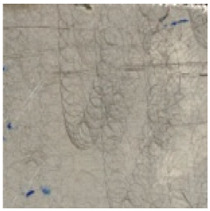	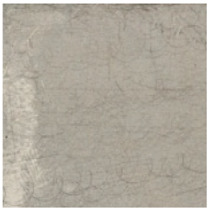
0°	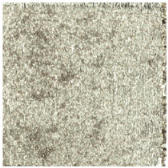	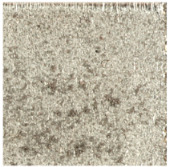	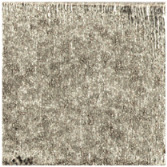
15°	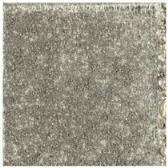	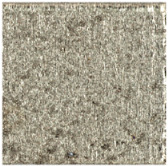	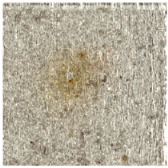
30°	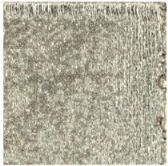	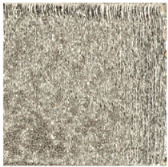	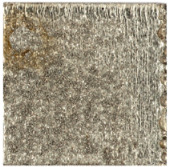
45°	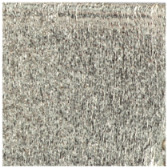	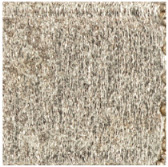	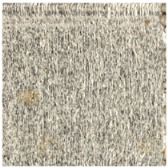
60°	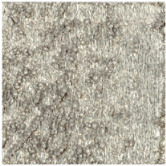	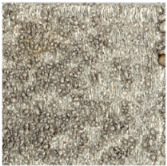	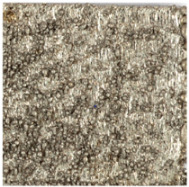

**Table 6:** Mass measurement results before and after long-term corrosion testing and corrosion rate values for individual groups of IN718 samples.

Temperature	Sample	Mass Before Corrosion Test [g]	Mass After Corrosion Test [g]	Mass Difference [g]	Mass Loss [%]	Corrosion Rate [g/cm^2^ × Year]
20 °C	Foil	2.7987	2.7978	0.0009	0.0322	0.0039
	0°	2.4479	2.4464	0.0015	0.0613	0.0066
	15°	2.4341	2.4335	0.0006	0.0246	0.0026
	30°	2.3616	2.3608	0.0008	0.0339	0.0035
	45°	2.2621	2.2617	0.0004	0.0177	0.0018
	60°	2.1530	2.1526	0.0004	0.0186	0.0018
45 °C	Foil	2.5564	2.5564	0.0000	0.0000	0.0000
	0°	2.4412	2.4400	0.0012	0.0492	0.0053
	15°	2.4579	2.4570	0.0009	0.0366	0.0039
	30°	2.3967	2.3964	0.0003	0.0125	0.0013
	45°	2.3136	2.3133	0.0003	0.0130	0.0013
	60°	2.1680	2.1672	0.0008	0.0369	0.0035

**Table 7:** Electrochemical parameters obtained during potentiostatic test.

Sample	Ecorr [mV]	Icorr [μA]
Foil	−1019	57
0°	−1133	29
15°	−1156	40
30°	−1203	59
45°	−1124	32
60°	−1166	49

**Table 8:** Images of the structures inclined at different angles before and after the corrosion process at 20 °C and 45 °C, taken on a scanning microscope (magnification ×50; the horizontal scale bar corresponds to 500 μm).

Sample	Before Corrosion Test	After Corrosion at 20 °C	After Corrosion at 45 °C
Foil	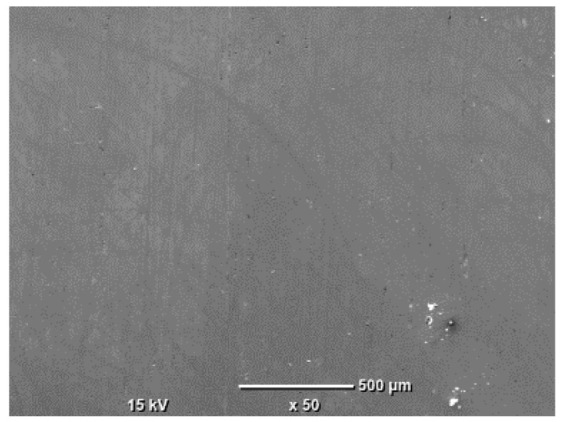	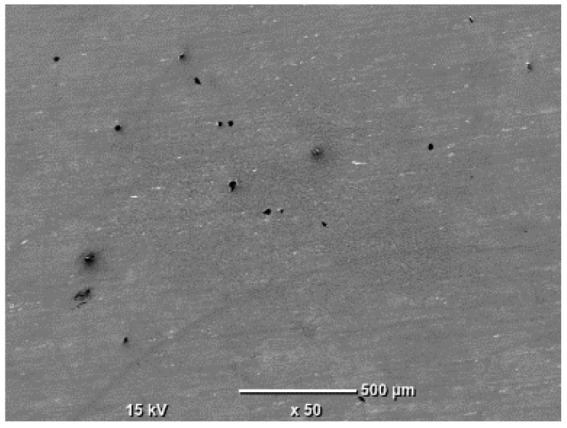	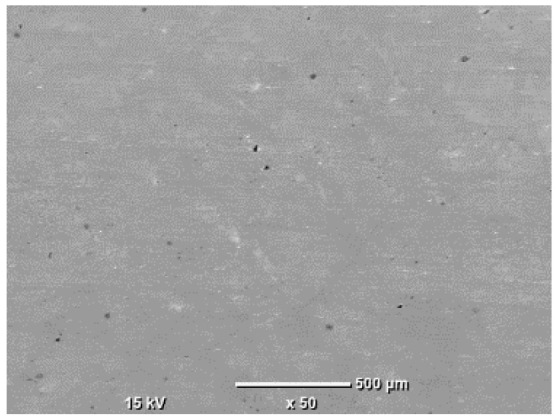
0°	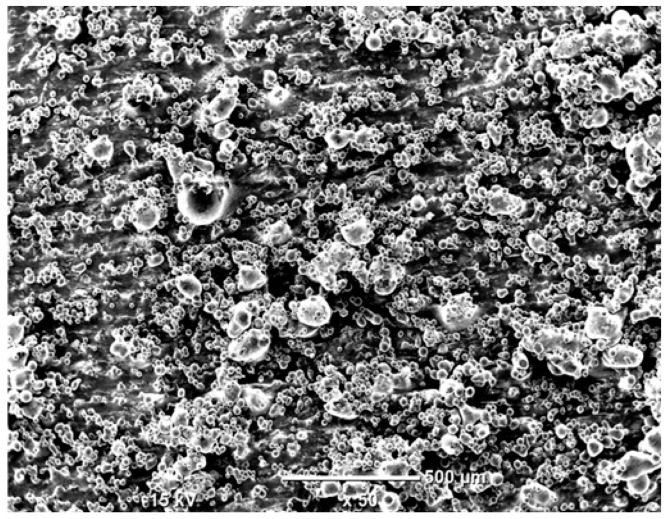	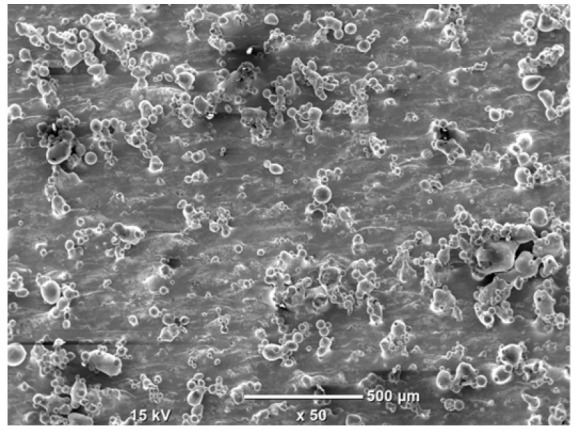	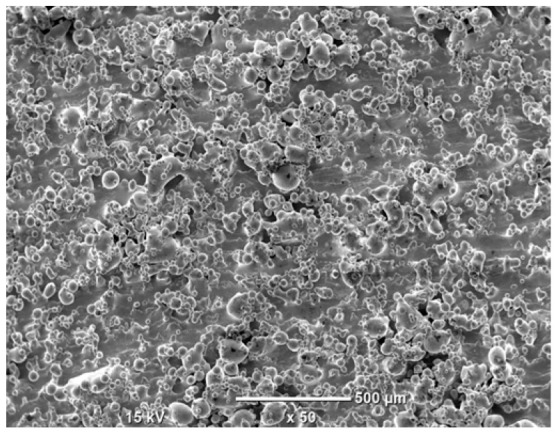
15°	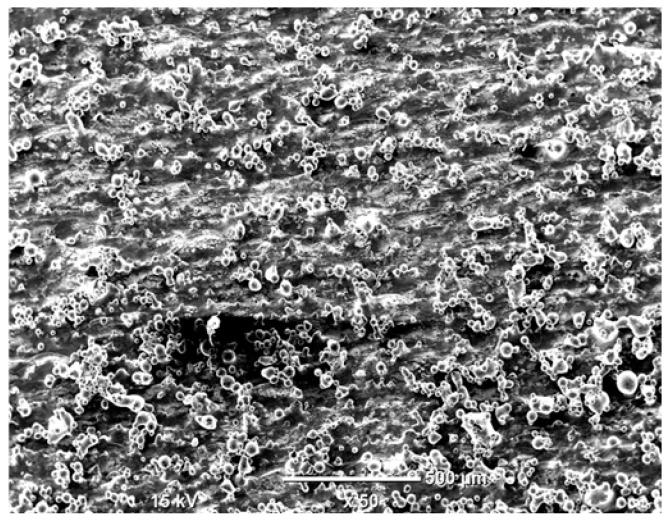	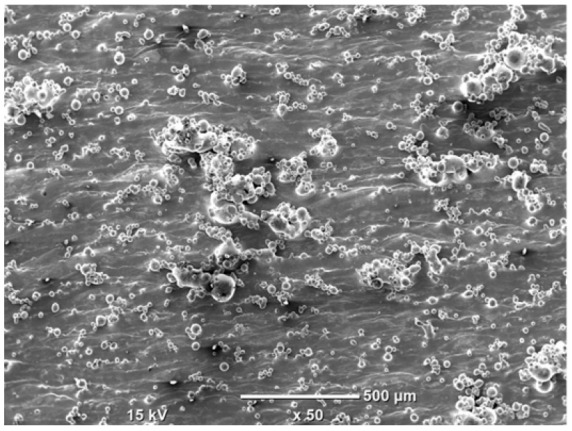	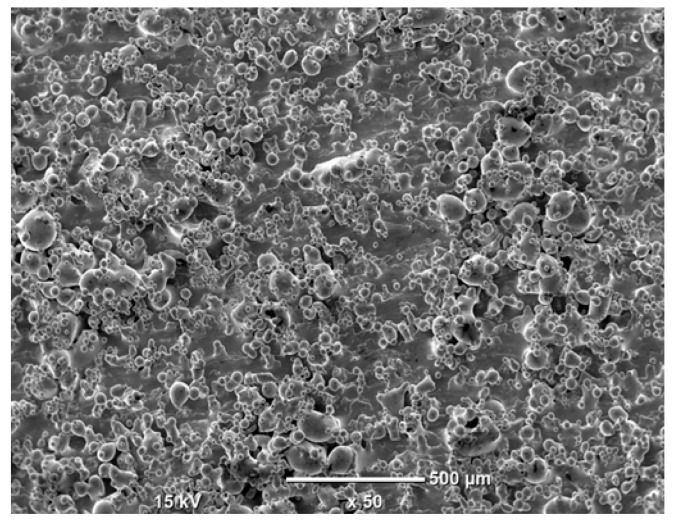
30°	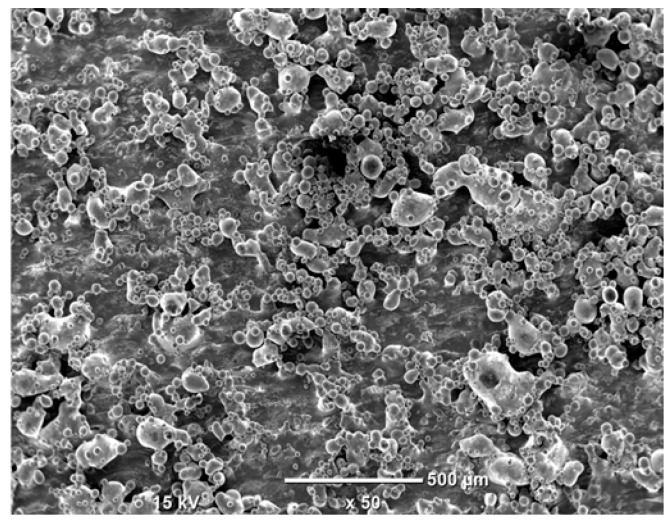	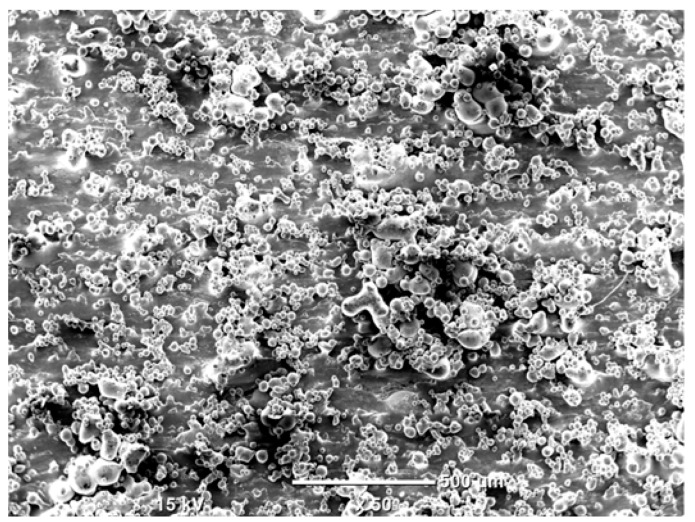	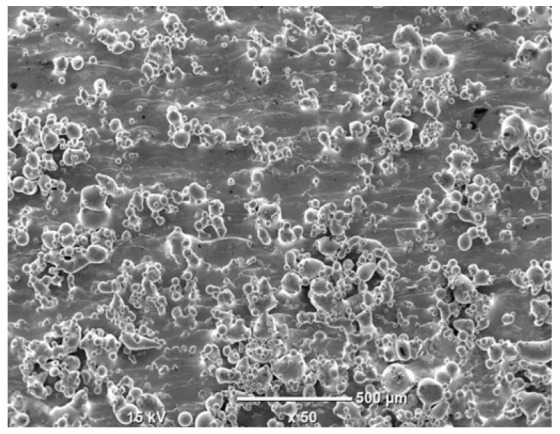
45°	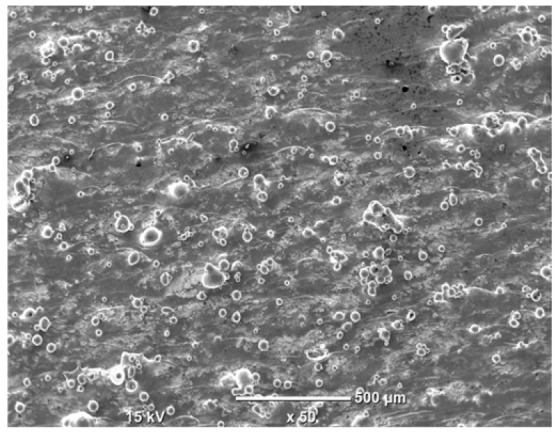	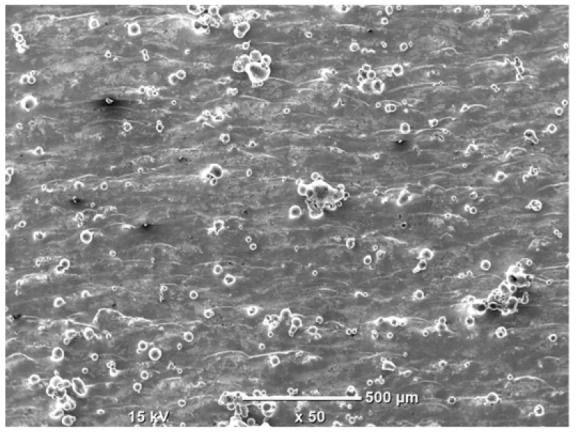	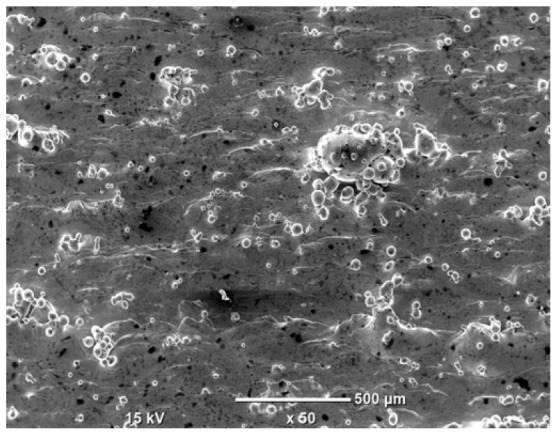
60°	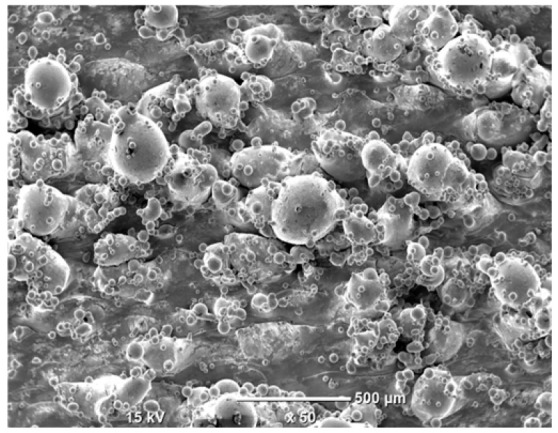	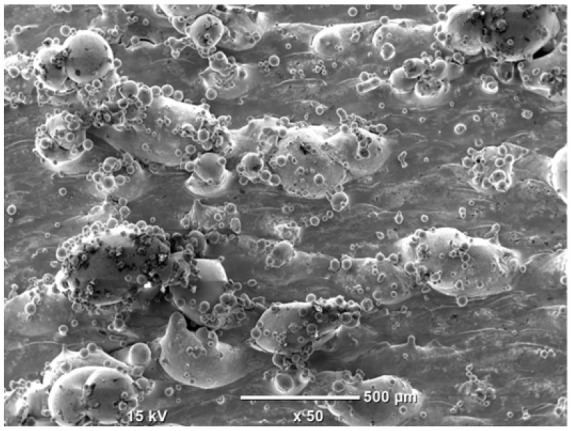	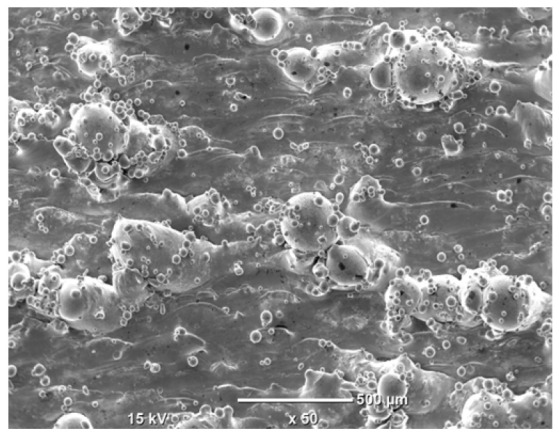

**Table 9:** EDS analysis results for surfaces subjected to long-term corrosion testing before corrosion testing, at room temperature, and at elevated temperature.

	Sample	Chemical Composition [at. %]								
		C	O	Al	Ti	Cr	Fe	Ni	Nb	Mo
Before corrosion	Foil	18.33	5.74	0.85	0.55	13.21	16.59	41.78	1.66	1.29
	0°	9.46	5.39	1.94	1.22	19.63	15.94	42.56	2.74	1.39
	15°	8.76	6.02	2.25	1.24	18.90	15.78	42.62	2.90	1.52
	30°	9.10	5.96	2.49	1.26	18.99	16.06	42.01	2.68	1.46
	45°	9.25	6.61	2.68	1.28	18.01	15.35	42.31	2.95	1.55
	60°	13.35	9.09	2.97	1.22	17.05	14.32	38.15	2.48	1.36
20 °C	Foil	18.37	5.48	0.97	0.56	13.84	17.28	43.51	0.00	0.00
	0°	7.05	5.89	2.26	1.30	18.83	15.90	44.08	3.10	1.60
	15°	9.19	4.90	1.65	1.13	19.02	16.14	43.77	2.78	1.44
	30°	12.43	6.43	2.14	1.21	18.20	15.42	40.19	2.59	1.39
	45°	7.13	6.42	2.63	1.25	18.74	16.02	43.19	3.13	1.50
	60°	16.89	10.79	3.17	1.21	15.86	13.32	34.95	2.53	1.29
45 °C	Foil	21.53	4.86	0.93	0.57	13.31	16.62	42.18	0.00	0.00
	0°	19.99	8.95	1.82	1.11	16.14	13.54	35.06	2.27	1.12
	15°	20.18	8.61	1.65	1.15	16.17	13.78	34.93	2.29	1.24
	30°	13.40	8.03	2.63	1.17	17.32	14.80	38.65	2.57	1.42
	45°	32.79	9.29	1.64	0.98	12.61	10.62	28.87	2.15	1.05
	60°	11.34	9.96	3.61	1.26	16.96	14.66	37.99	2.83	1.38

**Table 10:** Ra and Rz measurement results for the tested surfaces.

Corrosion Temperature	Sample	Ra_1_ [µm]	Ra_2_ [µm]	Ra_3_ [µm]	Ra [µm]	Rz_1_ [µm]	Rz_2_ [µm]	Rz_3_ [µm]	Rz [µm]
Before corrosion	Foil	198.68	193.48	191.19	194.45	6.44	8.87	14.69	10.00
	0°	21.34	25.48	22.44	23.09	176.05	174.58	173.58	174.74
	15°	27.84	30.97	27.23	28.68	179.72	217.49	194.82	197.34
	30°	19.32	22.32	19.90	20.51	154.51	196.86	160.75	170.71
	45°	5.88	11.83	6.43	8.05	89.58	170.25	104.02	121.28
	60°	36.55	91.59	54.42	60.85	387.97	472.98	343.23	401.39
20 °C	Foil	181.92	181.99	186.42	183.44	7.59	7.37	6.77	7.24
	0°	8.68	10.07	13.28	10.68	113.95	136.13	165.24	138.44
	15°	22.62	21.35	22.56	22.18	173.25	195.16	162.07	176.83
	30°	19.73	17.76	17.67	18.39	161.89	160.03	156.11	159.34
	45°	10.15	11.10	11.15	10.80	134.67	158.39	180.67	157.91
	60°	57.71	53.34	68.28	59.78	362.56	356.35	360.19	359.70
45 °C	Foil	159.69	163.51	157.94	160.38	6.95	4.14	6.42	5.84
	0°	19.97	18.51	20.95	19.81	161.79	142.02	160.50	154.77
	15°	27.06	28.82	30.37	28.75	173.85	191.87	178.41	181.38
	30°	31.01	28.48	25.51	28.33	189.08	172.16	175.52	178.92
	45°	10.00	7.41	8.36	8.59	132.68	110.78	129.23	124.23
	60°	75.56	40.91	45.67	54.05	384.00	308.88	264.06	318.98

**Table 11:** Surface topography of samples subjected to roughness testing.

Before corrosion—foil, Ra_1_ = 198.68 [µm], Rz_1_ = 6.44 [µm]
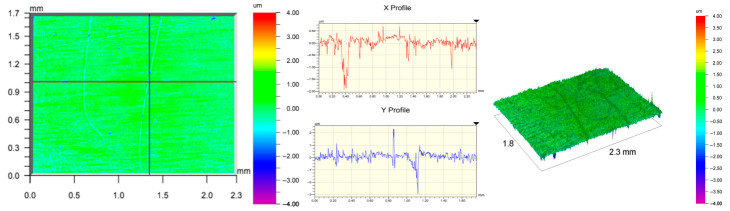
Corrosion at 20 °C—foil, Ra_1_ = 181.92 [µm], Rz_1_ = 7.59 [µm]
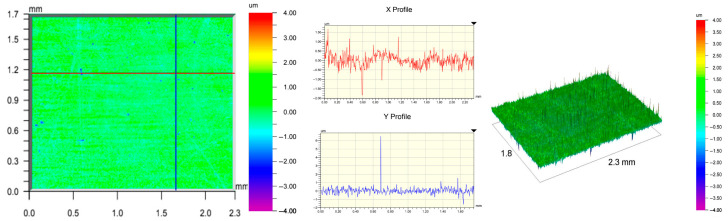
Corrosion at 45 °C—foil, Ra_1_ = 159.69 [µm], Rz_1_ = 6.95 [µm]
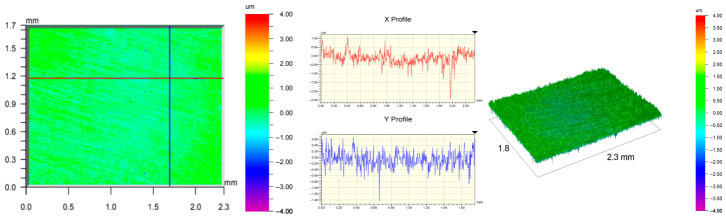
Before corrosion—angle 0°, Ra_1_ = 21.34 [µm], Rz_1_ = 176.05 [µm]
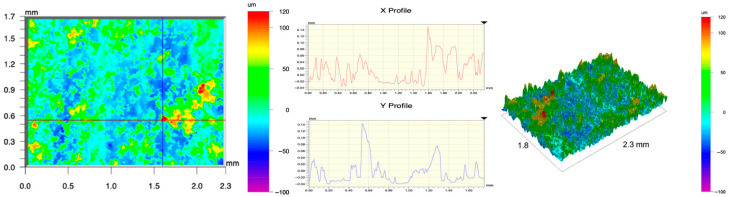
Corrosion at 20 °C—angle 0°, Ra_1_ = 8.68 [µm], Rz_1_ = 113.95 [µm]
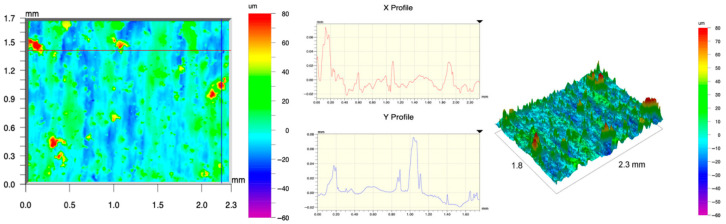
Corrosion at 45 °C—angle 0°, Ra_1_ = 19.97 [µm], Rz_1_ = 161.79 [µm]
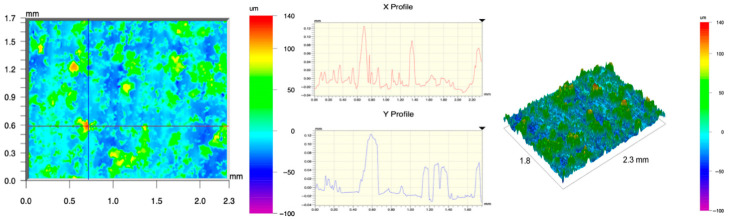
Before corrosion—angle 15°, Ra_1_ = 27.84 [µm], Rz_1_ = 179.72 [µm]
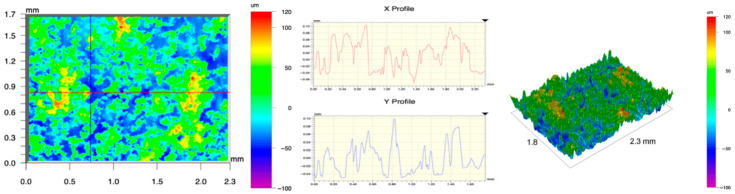
Corrosion at 20 °C—angle 15°, Ra_1_ = 22.62 [µm], Rz_1_ = 173.25 [µm]
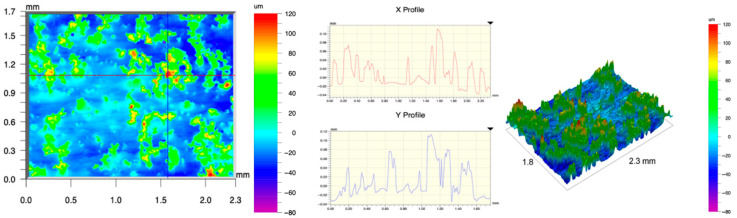
Corrosion at 45 °C—angle 15°, Ra_1_ = 27.06 [µm], Rz_1_ = 173.85 [µm]
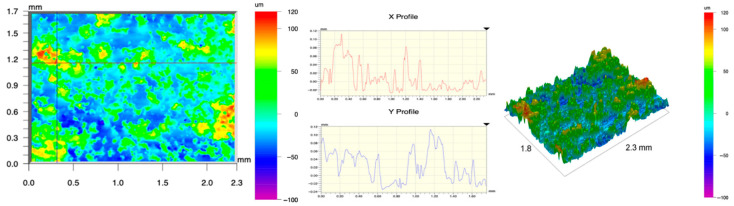
Before corrosion—angle 30°, Ra_1_ = 19.32 [µm], Rz_1_ = 154.51 [µm]
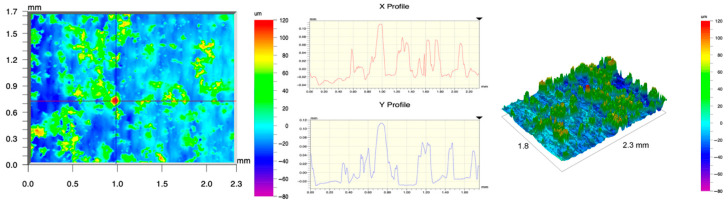
Corrosion at 20 °C—angle 30°, Ra_1_ = 19.73 [µm], Rz_1_ = 161.89 [µm]
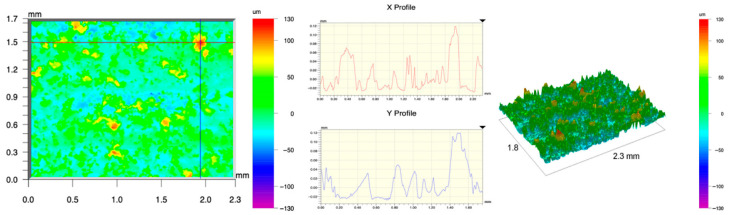
Corrosion at 45 °C—angle 30°, Ra_1_ = 31.01 [µm], Rz_1_ = 189.08 [µm]
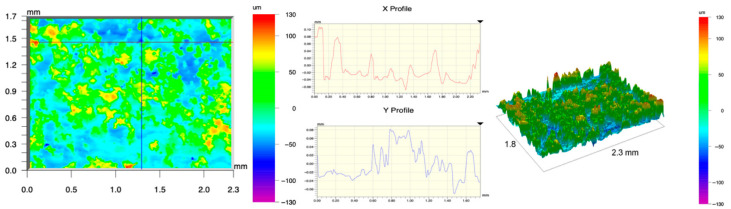
Before corrosion—angle 45°, Ra_1_ = 5.88 [µm], Rz_1_ = 89.58 [µm]
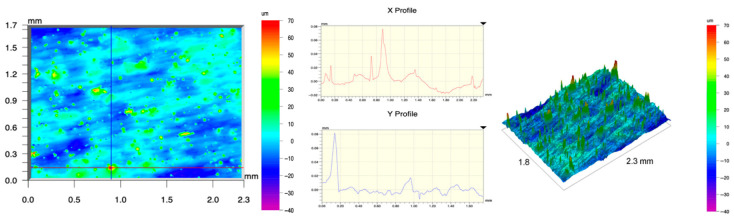
Corrosion at 20 °C—angle 45°, Ra_1_ = 10.15 [µm], Rz_1_ = 134.67 [µm]
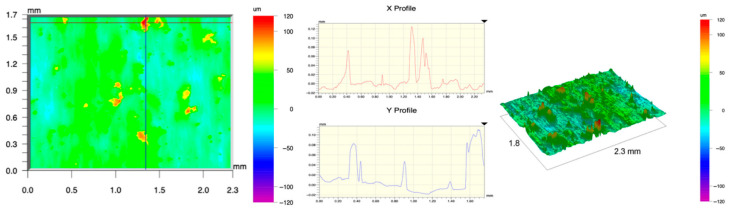
Corrosion at 45 °C—angle 45°, Ra_1_ = 10.00 [µm], Rz_1_ = 132.68 [µm]
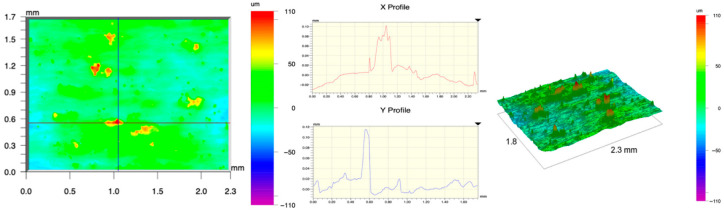
Before corrosion—angle 60°, Ra_1_ = 36.55 [µm], Rz_1_ = 387.97 [µm]
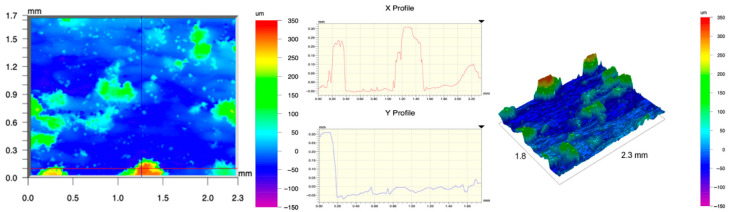
Corrosion at 20 °C—angle 60°, Ra_1_ = 57.71 [µm], Rz_1_ = 362.56 [µm]
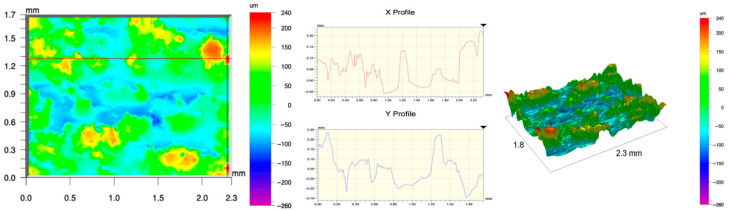
Corrosion at 45 °C—angle 60°, Ra_1_ = 75.56 [µm], Rz_1_ = 384.00 [µm]
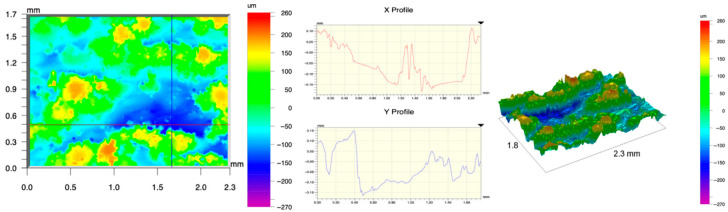

## Data Availability

The original contributions presented in this study are included in the article. Further inquiries can be directed to the corresponding author.
